# Statin prophylaxis and inflammatory mediators following cardiopulmonary bypass: a systematic review

**DOI:** 10.1186/cc8135

**Published:** 2009-10-20

**Authors:** Catherine Morgan, Michael Zappitelli, Peter Gill

**Affiliations:** 1 Division of Nephrology, Department of Pediatrics, University of Alberta, 2B2-42 WC Mackenzie Health Sciences Centre, Edmonton, Alberta, T6G 2R7, Canada; 2 Montreal Children's Hospital, McGill University Health Centre, 2300 Tupper Street, Montreal, Quebec, H3H 1P3, Canada; 3 Faculty of Medicine and Dentistry, University of Alberta, 2J2.00 WC Mackenzie Health Sciences Centre Edmonton, Alberta, T6G 2R7, Canada

## Abstract

**Introduction:**

Induction of an inflammatory response is thought to have a significant role in the complications that follow cardiopulmonary bypass (CPB). The statin drugs are increasingly being recognized as having potent anti-inflammatory effects and hence have potential to influence an important mechanism of injury in CPB, although there is no current confirmation that this is indeed the case. Our objective was to systematically review if pre-operative prophylactic statin therapy, compared with placebo or standard of care, can decrease the inflammatory response in people undergoing heart surgery with CPB.

**Methods:**

We performed a systematic and comprehensive literature search for all randomized controlled trials (RCTs) of open heart surgery with CPB in adults or children who received prophylactic statin treatment prior to CPB, with reported outcomes which included markers of inflammation. Two authors independently identified eligible studies, extracted data, and assessed study quality using standardized instruments. Weighted mean difference (WMD) was the primary summary statistic with data pooled using a random effects model. Descriptive analysis was used when data could not be pooled.

**Results:**

Eight RCTs were included in the review, with the number of trials for each inflammatory outcome being even more limited. Pooled data demonstrated benefit with the use of statin to attenuate the post-CPB increase in interleukins 6 and 8 (IL-6, IL-8), peak high sensitivity C-reactive protein (hsCRP), and tumor necrosis factor-alpha (TNF-α) post-CPB (WMD [95% confidence interval (CI)] -23.5 pg/ml [-36.6 to -10.5]; -23.4 pg/ml [-35.8 to -11.0]; -15.3 mg/L [CI -26.9 to -3.7]; -2.10 pg/ml [-3.83 to -0.37] respectively). Very limited RCT evidence suggests that prophylactic statin therapy may also decrease adhesion molecules following CPB including neutrophil CD11b and soluble P (sP)-selectin.

**Conclusions:**

Although the RCT evidence may suggest a reduction in post-CPB inflammation by statin therapy, the evidence is not definitive due to significant limitations. Several of the trials were not methodologically rigorous and statin intervention was highly variable in this small number of studies. This systematic review demonstrates that there is a significant gap that exists in the current literature in regards to the potential anti-inflammatory effect of statin therapy prior to CPB.

## Introduction

The use of cardiopulmonary bypass (CPB) is necessary for many cardiac surgical procedures. However, it is clear that CPB can have deleterious effects, including initiation of cardiopulmonary dysfunction, renal dysfunction, and neurological injury in the acute peri-operative period. Economic costs and the human costs are greater when cardiac surgical patients develop complications following CPB [1,2]. A number of different prophylactic strategies have been employed in attempts to improve clinical outcomes following CPB [3]. Previous systematic reviews and meta-analysis have looked at the effects of some of these interventions for preventing post-CPB organ dysfunction and there is no definitive evidence that these interventions are beneficial [4-6]. Thus, the development and use of new strategies aimed at reducing post-operative morbidity and mortality following CPB is of importance.

It is well documented that CPB is associated with a systemic inflammatory response, which involves the synthesis of various cytokines and inflammatory mediators [7]. This inflammation sets the stage for initiation of injury of major organs (including multiple organ dysfunction syndrome) as well as amplification of injury induced by alterations in vasoreactivity and organ perfusion [8]. Statins are being increasingly recognized as having pleotrophic (non-lipid mediated) effects, including inhibition of inflammation [9]. Given that statins can exert direct anti-inflammatory effects and what is understood about organ injury post-CPB, it is tempting to speculate that these drugs might have broad potential as an intervention in the pre-operative care of people undergoing CPB.

Previous reviews and meta-analyses that have looked at the clinical impact of pre-operative statin treatment on major adverse events after cardiovascular surgery have suggested a beneficial impact. For example, a systematic review by Liakopoulos and colleagues [10], which included 3 randomized controlled trials (RCTs), and 16 observational studies (3 prospective and 13 retrospective), reported outcomes of 31,725 cardiac surgery patients with or without pre-operative statin therapy. The meta-analysis provided evidence that pre-operative statin therapy exerts substantial clinical benefit on early post-operative adverse outcomes in cardiac surgery patients (including mortality, atrial fibrillation and stroke). A previous systematic review by Hindler and colleagues [11] had demonstrated a variable effect of peri-operative statin on post-operative cardiovascular morbidity. Given the limitations of the evidence base for these systematic reviews, the need for further RCTs was underscored by the investigators, with the empirical use of statins for all patients undergoing cardiac surgery not supported.

Understanding the biological mechanism by which statins may change the clinical course following cardiac surgery with CPB is important as we move forward in the development of large high-quality RCTs to further address the clinical efficacy of this intervention. It has not been demonstrated by systematic review that statins can modify the biological processes that are related to organ injury post-CPB. It is therefore prudent to systematically review this existing literature. The primary objective of this review was to answer the following question: in people undergoing heart surgery with CPB, does pre-operative prophylactic statin therapy, compared with placebo or standard of care, decrease the inflammatory response after CPB?

## Materials and methods

This systematic review was conducted and reported in accordance with available guidelines [12].

### Criteria for considering studies for this review

Criteria for types of studies were as follows: randomized clinical trials, with or without placebo. Studies were eligible if they included male or female patients of any age undergoing CPB for elective open-heart surgery (open-heart surgery could include surgery for coronary artery bypass, valve repair, or congenital heart lesions). Studies of patients undergoing heart transplant were excluded. Patients already on statins were excluded. Criterion for type of intervention was the pre-operative use of any statin drug administered as prophylaxis pre-CPB, with or without other interventions, compared with a non-statin containing control regimen (either standard of care or placebo). Co-interventions were allowed as long as all arms of the randomized allocation received the same co-interventions. The primary evaluation criterion for outcomes measures was the measurement of inflammatory markers post-operatively (e.g. interleukins (IL-6, IL-8, IL-1), TNF-α and its receptors, high sensitivity C-reactive protein (hsCRP), adhesion molecules (neutrophil CD11b, intercellular adhesion molecule-1 (ICAM-1), selectins), complement split products (C3a, C5a)).

### Search methods for identification of studies

We conducted a comprehensive search to identify all relevant studies of statin use. Cochrane Central Register of Controlled Trials (CENTRAL), MEDLINE (1950 to March 2009), EMBASE (1988 to March 2009), and International Pharmaceutical Abstracts (1970 to March 2009) were searched using relevant search terms relating to heart surgery procedures with CPB and statins [see Additional data file 1 for details of search strategies]. PubMed was searched for in-process records and other non-indexed citations. A number of clinical trials registers (n = 4) and a variety of meeting abstract (n = 5) and grey literature (Grey Literature Report) sources were searched. The citations of existing reviews and trials identified were reviewed to identify pertinent studies and articles citing the retrieved trials were identified by PubMed and via Web of Science.

For knowledge of ongoing or unpublished trials, we contacted investigators of all identified trials, a number of groups identified through the United States National Library of Medicine's Directory of Health Organizations and the Cochrane Collaborative Review Groups' Specialized Register, and pharmaceutical companies. Studies in all languages were included.

### Data collection and analysis

#### Selection of trials

**Figure 1:**
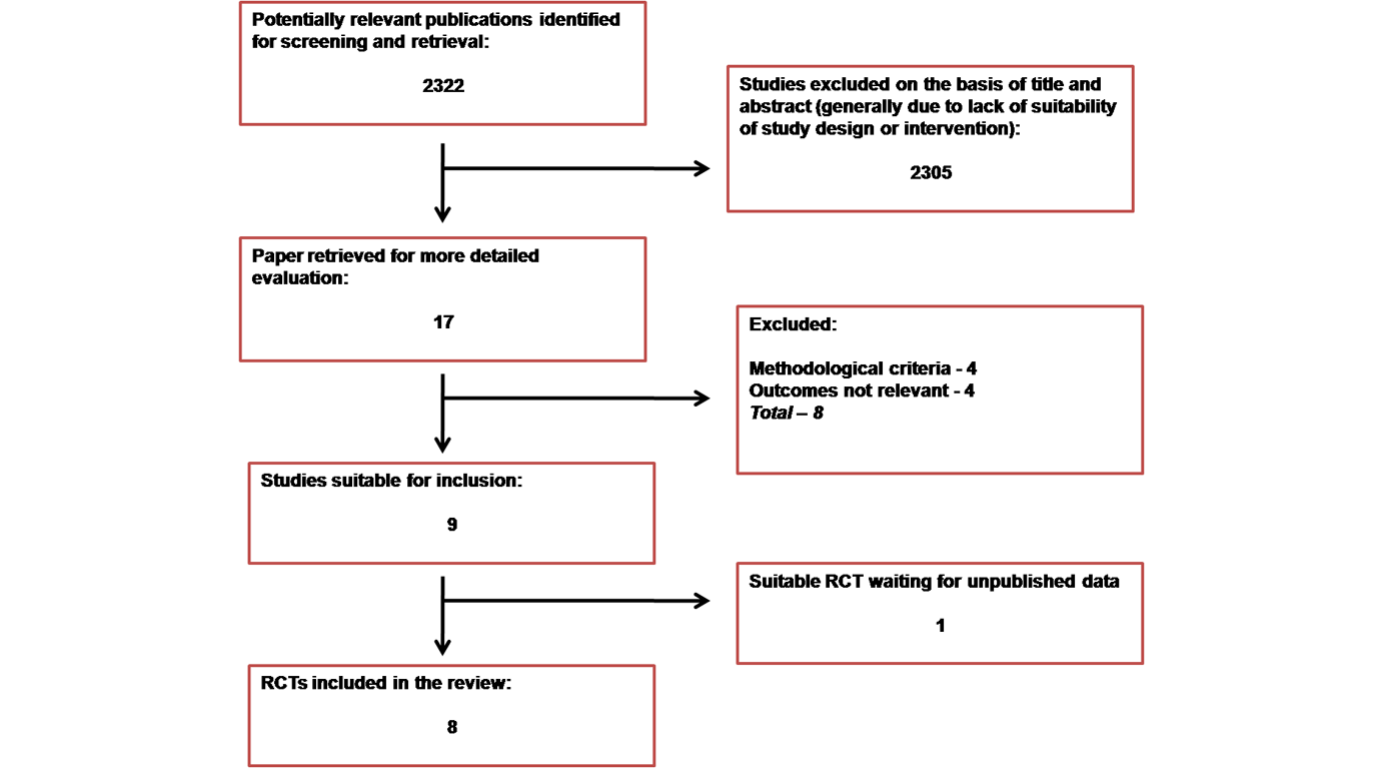
Flowchart for selection of randomized control trials.  RCT = randomized controlled trial.

Two authors (CM and either MZ or PG) independently assessed titles and abstracts identified from the electronic database searches, applying predetermined eligibility criteria. If two reviewers were certain that a reference was not relevant it was excluded. Disagreements between authors were solved by consensus. Full text of articles that were judged to be potentially eligible by title and abstract review as above were retrieved and two reviewers (CM and MZ) assessed them for eligibility using the inclusion and exclusion criteria. If both were certain that a study was unsuitable it was excluded and reasons for exclusion noted. Disagreements between authors were solved by consensus. The reviewers were not blinded for authors' names or journal names. The numbers of references retrieved from the searches was recorded. Those eligible or ineligible were documented and a quality of reporting of meta-analysis (QUOROM) statement prepared [12] (Figure 1).

#### Assessment of methodological quality of included studies

Quality of the studies was assessed by two reviewers (CM and either MZ or PG) without blinding to the journal or authorship, using the established standards of the Cochrane Collaboration (Cochrane collaboration Risk of Bias Assessment Tool) [13]. The quality items assessed were sequence generation, allocation concealment, blinding of participants, personnel and outcome assessors, completeness of outcome data, selective outcome reporting, and other sources of bias. Each item was assessed as adequate (i.e. low risk of bias), inadequate (high risk of bias), or unclear (uncertain risk of bias). For detailed description of this tool for assessing risk of bias see Table 8.5a in the Cochrane Handbook [13]. The methodological quality of the selected studies was scored overall by a summary assessment of the risk of bias; low, unclear, and high risk were the three possible categories. Studies judged as low risk of bias scored as adequate for all key items. Studies judged as unclear risk of bias scored as unclear for one or more key items. Studies judged as high risk of bias scored as inadequate for one or more key items. If information was not available in the trial reports necessary for risk of bias assessment, further information was sought by correspondence with the principal investigator(s). Discrepancies between reviewers were resolved by discussion.

#### Data extraction for articles meeting the inclusion criteria

Data was extracted independently by two reviewers (CM and MZ) and was collected on a data extraction form, which had been pre-tested to ensure appropriate data collection. This form included basic identifying information, information on trial quality, and information on trial participants, interventions, and outcomes related to post-operative inflammatory markers. Data regarding the clinical endpoints of mortality and morbidity was also extracted.

#### Statistical methods

Data were analyzed using Review Manager (RevMan) 5 for analysis. For continuous variables (e.g. levels of inflammatory markers) weighted mean difference (WMD) with 95% confidence interval (CI) was used when combining data. Data was pooled where appropriate using random effects models and statistical heterogeneity was quantified using the *I*^2 ^statistic [14].

## Results

### Description of studies

Of the 2322 titles and abstracts found, 17 studies were identified as potentially suitable and the full text retrieved (Figure 1). Fifteen of these studies were English language; the other two studies (one Chinese and one Spanish with the abstracts in English) were translated to allow classification and further analysis. Of the 17 studies, we included 8 studies in the review and excluded 9 studies. One of the nine excluded studies is eligible for inclusion (Nakamura and colleagues [15]); however, only a subgroup of the study sample had CPB and this data is not available in published form (we have attempted to contact the author with no response and hence have not included it further in the review). Four excluded studies did not fulfill the methodological criteria because they were non-randomized, prospective cohort studies [16-19]. Four studies were excluded due to type of outcome measures [20-23]. A breakdown of each of the excluded studies, as well as the study by Nakamura and colleagues [15], addressing the specifics of their study design and outcomes can be found in Additional data file 2: characteristics of excluded studies, studies awaiting assessment, and ongoing studies. We obtained all of the included studies as a result of the database searches. With the exception of Tamayo and colleagues [24], we have had limited success in receiving adequate information and feedback from the authors that we have attempted to contact, in spite of repeated attempts. Details of included studies are given in Table 1. We identified two ongoing RCTs fulfilling eligibility criteria. One was identified through the Australian New Zealand Clinical Trials Registry with registration number ACTRN12606000405516 [25] and the other was through ClinicalTrials.gov, a service of the U.S. National Institutes of Health, with registration number NCT00791648 [26]. Details are given in Additional data file 2: characteristics of excluded studies, studies awaiting assessment, and ongoing studies. All of the included and excluded studies were published between 2001 and 2009.

**Table 1:** Characteristics of included studies

Trial	Methods	Participants	Interventions	Outcomes
**Berkan et al. **[28]	Randomized but method not clear; unclear allocation concealment but adequate blinding; unclear risk of bias.	CABG with CPB. Statin group n = 23, age, mean = 65.4, SD 11.2; control group n = 23, age, mean 67.7, SD 9.6. Statin group duration of CPB, mean 122.4, SD 36.9; control group duration of CPB, mean 113, SD 27.2.	Fluvastatin 80 mg daily for 3 weeks before CPB. Control group received placebo in same manner.	sP-selectin level; clinical outcomes (inotrope use, length of ICU and hospital stay, incidence of MI).
				
**Caorsi et al. **[29]	Randomized but method not clear; unclear allocation concealment and not blinded; high risk of bias.	CABG with CPB. Statin group n = 21, age, mean = 68.2, SD 7.2; control group n = 22, age, mean 67.9, SD 7.3. Statin group duration of CPB, mean 93.8, SD 9.1; control group duration of CPB, mean 94.1, SD 7.7.	40 mg pravastatin daily from 48 hours prior to CPB to post-operative day 7; additional dose 1 hour after CPB. Control group received standard of care with no placebo. Both groups received aspirin 6 hours after CPB.	Inflammatory cytokines
				
**Chello et al. (2006) **[30]	Randomized but method not clear; unclear allocation concealment but adequate blinding; unclear risk of bias. a	CABG with CPB. Statin group n = 15, age, mean = 65.7, SD 7.7; control group n = 15, age, mean 63.7, SD 7.1. Statin group duration of CPB, mean 97, SD 5.5; control group duration of CPB, mean 94.3, SD 8.6.	Atorvastatin 20 mg daily for 3 weeks before CPB. Control group received placebo in same manner.	Inflammatory cytokines; neutrophil adhesion and function; endothelial nitric oxide release; SIRS
				
**Chello et al. (2007) **[27]	Randomized but method not clear; unclear allocation concealment but adequate blinding; unclear risk of bias.	CABG with CPB. Statin group n = 15, age, mean = 67.7, SD 6.2; control group n = 15, age, mean 66.3, SD 7.5. Statin group duration of CPB, mean 97.9 SD 19.4; control group duration of CPB, mean 102.5, SD 28.2.	Simvastatin 40 mg daily starting 3 weeks prior to CPB. Control group received placebo in same manner.	Inflammatory cytokines; neutrophils apoptosis and function.
				
**Florens et al. **[33]	Randomized but method not clear; unclear allocation concealment; not blinded; some patients received aprotinin, although indications not given; high risk of bias. b	Heart surgery with CPB. Statin group n = 10, age, mean = 68, SD 18; control group n = 10, age, mean 62, SD 12. Statin group duration of CPB, mean 89, SD 24; control group duration of CPB, mean 93, SD 35.	Atorvastatin 40 mg 18 hrs pre-operatively and 40 mg immediately pre-operatively. Control group received standard of care with no placebo.	Inflammatory cytokines;atrial biopsy for nuclear factor kappa B; clinical outcomes (ventilation time, fever, leukocytosis, renal dysfunction, MI, inotrope use)
				
**Mannacio et al. **[31]	Randomized; allocation concealed and adequate blinding; low risk of bias.	CABG with CPB. Statin group n = 100, age, mean 61.3, SD 9.2; control group n = 100, age, mean 59.3, SD 8.4. Statin group duration of CPB, mean 80.6, SD 22.4; control group duration of CPB, mean 83.8, SD 25.2	Rosuvastatin 20 mg daily starting 7 days before CPB; Control group received placebo in same manner.	hsCRP; myocardial damage; atrial fibrillation; low output syndrome; renal failure
				
**Patti et al. **[32]	Randomized; allocation concealed and adequate blinding; low risk of bias.	Heart surgery with CPB (CABG, valve repair, aortic aneurysm repair). Statin group n = 101, age, mean 65.5, SD 8.8; control group n = 99, age, mean 67.3, SD 8.1. Statin group duration of CPB, mean 113, SD 37; control group duration of CPB, mean 105, SD 30.	Atorvastatin 40 mg daily for 7 days before CPB; continued day after surgery until discharge. Control group received placebo in same manner.	Post-operative atrial fibrillation; length of post-operative hospital stay; major cardiac/cerebrovascular adverse events; hsCRP levels.
				
**Tamayo et al. **[24]	Randomized but method not clear; unclear allocation concealment; state that except for perfusionist, no member of medical team knew what group patient was randomized to; however there was no placebo given. Blinding of investigators is unclear. c	CABG with CPB. Statin group n = 22, age, mean 67.7, SD 7.3; control group n = 22, age, mean 68, SD 6.9. Statin group duration of CPB, mean 106.8, SD 26.9; control group duration of CPB, mean 96.2, SD 24.6.	Simvastatin 20 mg/day for 3 weeks before surgery versus no pre-operative simvastatin treatment.	hsCRP; IL-6; C4; clinical outcomes (renal dysfunction, ventilation).

CABG = coronary artery bypass graft; CPB = cardiopulmonary bypass; C4 = complement component 4; hsCRP = high sensitivity C-reactive protein; ICU = intensive care unit; IL-6 = interleukin-6; MI = myocardial infarction; SD = standard deviation; SIRS = systemic inflammatory response score; sP-selectin = soluble P-selectin

^a ^No response to letter requesting details.

^b ^Statistical tests requiring the assumption of normal distribution were used to detect between-group differences in cytokine levels (despite small sample size and no confirmation that data was indeed normally distributed, graphical representation suggests that data is likely skewed).

^c ^Data in the original paper is presented in graphical form. The author was contacted and provided exact point estimates and variability of the data which was not included in the published form.

The eight included studies comprised a total of 638 patients; 312 of these received prophylactic statin treatment prior to CPB and 296 acted as controls. The 2007 study by Chello and colleagues [27] has three study arms; one group received statin pre-operatively before CPB, one group received a placebo before CPB and a third group served as a control group undergoing surgical intervention but not CPB. We have excluded the third arm of the study. A total of 15 patients were excluded from the review due to this process. We were then left with a total of 623 patients, 312 of whom received statin treatment and 311 who acted as controls. All selected studies involved adult populations. Out of 312 patients receiving statins, 238 were male and 225 out of 311 patients acting as controls were male. Six trials were performed in patients undergoing coronary artery bypass graft (CABG) surgery [24,27-31] and two trials [32,33] were performed in patients undergoing various cardiac operations that required CPB (CABG, valve replacements and aortic aneurysm repair).

Various statin treatments were used in the different trials. Two trials used simvastatin: one trial used 40 mg/day [27] and the other used 20 mg/day [24]. Three trials used atorvastatin: one trial used 40 mg/day [32], one trial used 20 mg/day [30], and one trial used 40 mg twice per day [33]. Rosuvastatin was used in one trial at a dose of 20 mg/day [31]. Fluvastatin was used in one trial at a dose of 80 mg/day [28]. Pravastatin was used in one trial at a dose of 40 mg/day [29]. The duration of administration prior to CPB also varied between trials. Four trials administered treatment for three weeks before surgery [24,27,28,30], two trials administered treatment for one week before surgery [31,32], one trial administered treatment for two days before surgery [29], and one trial administered treatment one day before surgery [33].

### Risk of bias in included studies

**Figure 2:**
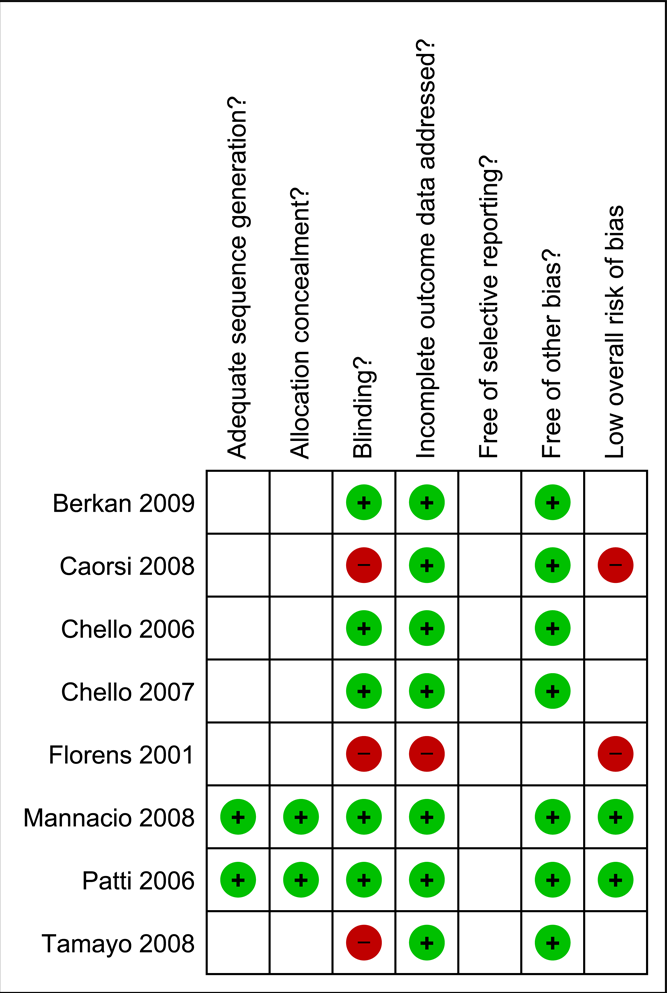
Methodological quality summary.  Review authors' judgements about each methodological quality item for each included study; light grey circle/(+) indicates adequate; dark grey circle (-) indicates inadequate; blank box indicates unclear.

Overall, study quality was not high, with only two studies having low risk of bias [31,32], four studies having unclear risk of bias [24,27,28,30], and two studies with high risk of bias [29,33] (Figure 2). All of the trials, with the exception of two, were small [31,32]. Three studies [28,30,32] discussed sample size determination or whether the study was adequately powered to demonstrate significance, in relation to the primary outcomes of interest for this systematic review. Authors were contacted for further methodological detail, but no additional information was provided. The authors of two studies explicitly stated no disclosures [30,32]. None of the publications mentioned any conflict of interest with respect to the drugs used and hence we assumed that none existed.

### Effects of interventions

With the exception of CD11b and hsCRP in one study [31], no studies reported change scores before and after CPB, although all studies measured pre-operative and post-operative levels of inflammatory markers. All of the studies in the review demonstrated no difference between groups for baseline measure of inflammatory markers prior to CPB. The only outcome demonstrating heterogeneity in pooled analysis by *I*^2 ^statistic was hsCRP; *I*^2 ^= 0 for all other pooled outcomes.

#### IL-6

Five of the included studies with 175 participants reported post-operative IL-6 level [24,27,29,30,33]. All five RCTs specified IL-6 as a primary outcome. The type of statin used varied (atorvastatin in two trials, simvastatin in two trials and pravastatin in one trial) and duration of pre-operative therapy ranged from 18 hours to 3 weeks. Only four of these studies were pooled, because we were unclear if the report by Florens and colleagues [33] provided the necessary data to be included in the pooled comparison; we suspect the data is skewed and it is unclear how the data is represented.

**Figure 3:**
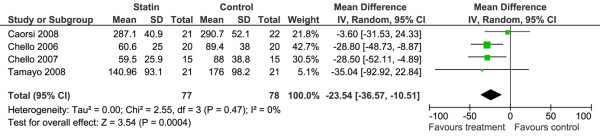
Forest plot of comparison for prophylactic statin therapy versus standard care/placebo: Inflammatory markers, outcome: IL-6.  Total refers to number of patients per trial. CI = confidence interval; SD = standard deviation.

The pooled data (Figure 3), which equates to a total of 155 participants, demonstrated benefit with the use of statin to reduce the post-operative peak level of IL-6 (WMD -23.5 pg/ml, 95% CI -36.6 to -10.5) measured at four to six hours post-CPB. Three of the pooled studies were of unclear risk of bias and one was high risk of bias [29].

Although the study by Florens and colleagues [33] reports that they failed to demonstrate this benefit, graphic representation of the data shows a point estimate of the four hour post-CPB IL-6 level in the statin group that is 25% of the four hour post-CPB IL-6 level in the control group.

#### IL-8

**Figure 4:**
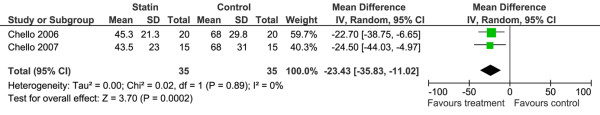
Forest plot of comparison for prophylactic statin therapy versus standard care/placebo: Inflammatory markers, outcome: IL-8.  Total refers to no. of patients per trial. CI = confidence interval; SD = standard deviation.

Three of the included studies with 80 participants reported post-operative IL-8 level [27,30,33]. All three RCTs specified IL-8 as a primary outcome. Two trials used atorvastatin and one trial used simvastatin. Duration of pre-operative therapy ranged from 18 hours to 3 weeks. Again, only two of the studies were pooled because we were unclear of the distribution and data reporting in the study by Florens and colleagues [33]. Both of the studies pooled came from the same investigative group and were both judged to be of unclear risk of bias. The pooled data (Figure 4) demonstrated benefit with the use of statin to reduce the post-operative level of IL-8 (WMD -23.4 pg/ml, 95% CI -35.8 to -11.0), measured four hours post-CPB. Florens and colleagues [33], which was judged to be of high risk of bias, reported failure to demonstrate this benefit, although the point estimate of the four-hour post-CPB IL-8 level in the statin group is lower than the four-hour post-CPB IL-8 level in the control group estimate for post bypass level.

#### hsCRP

**Figure 5:**
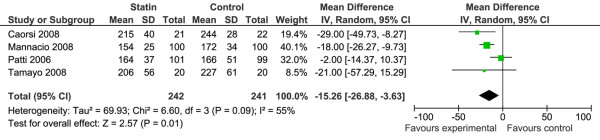
Forest plot of comparison for prophylactic statin therapy versus standard care/placebo: Inflammatory markers, outcome: hsCRP.  Total refers to no. of patients per trial. CI = confidence interval; SD = standard deviation.

Four studies with 487 participants reported post-operative hsCRP [24,29,31,32]. Two RCTs specified hsCRP as a primary outcome [24,29]. Each trial used a different statin (pravastatin, simvastatin, rosuvastatin, atorvastatin). Duration of pre-operative therapy ranged from 48 hours to 3 weeks. Two studies were low risk of bias, one study was unclear risk of bias, and one study was high risk of bias. The pooled analysis (Figure 5) demonstrated benefit with the use of statin to reduce the post-operative peak level of hsCRP (WMD -15.3 mg/L, 95% CI -26.9 to -3.7). Each trial measured hsCRP at 24-hour intervals post-CPB to a minimum of 48 hours and reported 'peak' levels; however, only two trials [24,29] specified the time at which this peak occurred (48 hours post-CPB). The percent of variance due to between-study variance was moderate to substantial (*I*^2 ^= 55%). The two individual studies judged to be low risk of bias and with significantly larger number of participants in each (n = 200) show discrepant results. Patti and colleagues [32] demonstrated that peak hsCRP levels after the operation did not differ between treatment and control groups (164 ± 37 versus 166 ± 51 mg/L, *P *= 0.75). In Mannacio and colleagues [31], mean post-operative peak hsCRP level was significantly lower in the treatment group compared with the control group (154 ± 2.5 mg/L versus 172 ± 3.4 mg/L, *P *< 0.001). Neither of these studies specified at which time point peak hsCRP was measured. The duration of pre-operative statin therapy was the same in both studies (1 week), although they differed in terms of the statin used (rosuvastatin [31] and atorvastatin [32]). Participants in both studies were predominantly undergoing CABG; however, Mannacio and colleagues [31] excluded patients undergoing additional cardiac surgery while Patti and colleagues [32] included a population having valve repair. Mean time on CPB was longer in the trial by Patti and colleagues [32].

#### TNF-α

**Figure 6:**
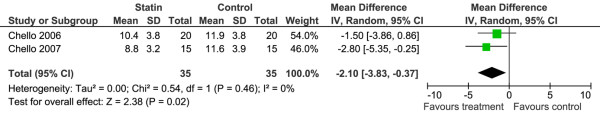
Forest plot of comparison for prophylactic statin therapy versus standard care/placebo: Inflammatory markers, outcome: TNF-α.  Total refers to no. of patients per trial. CI = confidence interval; SD = standard deviation.

Three of the included studies with 103 participants measured post-operative TNF-α level [27,29,30]. All three RCTs specified IL-8 as a primary outcome. One trial used atorvastatin, one trial used simvastatin, and one trial used pravastatin. Duration of pre-operative therapy ranged from 48 hours to 3 weeks. Caorsi and colleagues [29] present no data on the TNF-*α *level; they state that both groups showed low levels throughout the entire study; however, the detection limit of their technique was 10 pg/ml which is significantly higher than the detection limit in other included studies. The pooled data of two studies [27,29,30] (70 participants), demonstrated benefit with the use of statin to reduce the post-operative level of TNF-α (WMD -2.10 pg/ml, 95% CI -3.8 to -0.4) (Figure 6). Both of the studies pooled came from the same investigative group and were both judged to be of unclear risk of bias.

#### Adhesion molecules

##### CD11b

Three of the included studies with 80 participants reported neutrophil CD11b expression [27,30,33]. Two trials used atorvastatin and one trial used simvastatin. Duration of pre-operative therapy ranged from 18 hours to 3 weeks. All three studies specified CD11b expression as a primary outcome.

Florens and colleagues [33] measured CD11b expression by neutrophils isolated from blood drawn at the beginning and end of CPB. They report that post-CPB values of expression were significantly higher than pre-CPB expression and that statin had no effect on this change from baseline, although no statistical analysis for this is published.

The 2006 study by Chello and colleagues [30] also measured CD11b expression in neutrophils isolated from blood drawn at 0 (at CPB termination), 4 and 12 hours post-CPB; statin treatment significantly decreased the percent increase in CD11b expression relative to baseline at 4 and 12 hours (mean difference of -63%, 95% CI -92% to -34% at 4 hours and mean difference of -47%, 95% CI -71 to -22 at 24 hours).

The 2007 study by Chello and colleagues [27] measured expression by neutrophils isolated from blood drawn at the end of bypass and cultured for 8, 12, and 24 hours and report that CD11b expression was significantly higher (*P *< 0.01) in samples from the placebo group (145% increase in expression) compared with the statin group (102% increase in expression) after 24 hours of culturing.

##### ICAM-1

One of the studies, judged as high risk of bias, reported the effect of statin on the soluble form of the endothelial ICAM-1 [33]. The level measured 24 hours post bypass did not differ between statin and control groups (294 ± 70 versus 258 ± 94 ng/ml, *P *= 0.34)

##### P-selectin

Two studies describe measuring P-selectin levels following CPB [28,33], Florens and colleagues [33] report that post-operative values were not different between statin (n = 10) and control (n = 10) groups for any of the tested markers but do not provide data. This study was judged as high risk of bias. In the study by Berkan and colleagues [28], peak P-selectin levels were significantly different between statin (n = 23) and control (n = 23) (164.77 ± 15.5 ng/ml versus 260 ± 9.98 ng/ml, *P *< 0.001)). This study was judged as unclear risk of bias.

## Discussion

Statins are beginning to be used in a number of clinical situations where inflammation is considered a major pathophysiological mechanism including in people undergoing CPB. Pooled data in this systematic review demonstrates that statin therapy before CPB is associated with a reduction in circulating markers of inflammation, specifically IL-6, IL-8, hsCRP, and TNF-α. For IL-6 and IL-8, one additional trial, which was not pooled due to limitations of the original data, illustrates a 75% reduction in IL-6 with statin given pre-CPB and a 20% reduction in IL-8 with statin given pre-CPB. These reductions in inflammatory markers were not reported as significant in the original article; however, the sample size was very small. For TNF-α, un-pooled data which represents one additional study suggests that both treatment and control groups had similar levels of TNF-α throughout the study, although they were all below the detection limit for their assay. In addition, the detection limit of the assay used was not adequate, given the mean for TNF-α concentrations in other studies included in the review.

For IL-8 and TNF-α, pooled analysis was limited to only two studies, both of which came from the same investigative group [27,30]; no heterogeneity was detected between these two studies in pooling for either of these outcomes, which is not surprising given similar trial characteristics. It is of interest that the type of statin used was different between these trials (atorvastatin [30] and simvastatin [27]), which might suggest drug type is not an important determinant of between study differences. For IL-6, although our conclusions from pooled analysis are based on only four RCTs with different drug interventions, they are somewhat strengthened by the homogeneity of study results. Although pooled analysis detected a benefit of treatment with statin for reducing peak hsCRP level, there was moderate to substantial heterogeneity. Given the small number of included trials, subgroup/sensitivity analysis could not investigate potential between study differences. In addition, the two high-quality studies with larger number of participants demonstrated differing results, the reason for which is not clear.

Prophylactic statin therapy may decrease adhesion molecules following CPB including neutrophil CD11b and soluble P (sP)-selectin, although the evidence for this is weak. Neutrophil CD11b surface adhesion molecule expression is a marker of neutrophil stimulation. Although statin does not appear to change expression immediately following CPB, other data supports an effect of statin on neutrophils when assessed after a period of time more appropriate for protein transcription to occur.

Seven of the eight studies identified for inclusion in this systematic review measured clinical outcomes [24,28-33]. For the majority of the studies, clinical endpoints were not identified as primary outcomes. Given the small number of participants in these studies, the rarity of the events, and the limited numbers of trials to include in meta-analysis, we have not reported detailed results for these outcomes. In light of the fact that we did not systematically search for these outcomes, limited conclusions can be made. Previous systematic reviews have shown the beneficial effect of pre-operative statin treatment on major adverse cardiac outcomes including atrial fibrillation [10,11]. Four of the studies included in this review measured risk of atrial fibrillation [29-32] (483 participants, event rate 34%) and pooled analysis demonstrated benefit with the use of pre-operative statin to reduce atrial fibrillation post-operatively (risk ratio (RR) 0.58, 95% CI 0.45 to 0.75, risk difference (RD) -0.19, 95% CI -0.27 to -0.10, number needed to treat (NNT) 5, 95% CI 4 to 10). Statins also appeared to decrease the risk of inotrope use (4 studies [28,30-32], 486 participants, event rate 23%, RD -0.08, 95% CI -0.21 to -0.04, NNT 12, 95% CI 5 to 25).

Overall this review suggests that several markers of the inflammatory response post-CPB may be attenuated by pre-operative statin therapy. The findings of this review and the strength of the conclusions are limited by several factors related to the current state of evidence; the total number of studies is limited, the trial size is generally small, and the methodological quality of trials is generally not high. Although key investigators in this field who were contacted did not reveal any unpublished data, an additional limitation to this systematic review is that we did not statistically evaluate publication bias; the number of identified trials was small and the utility of such tests to assess the possibility of bias in this situation is extremely limited.

A meta-analysis of similar, well-conducted, RCTs is considered one of the highest levels of evidence but the primary trials all have to be conducted with high methodological rigor for the meta-analysis to be definitive. This is not the case for the evidence summarized in this review. In addition, controversy also arises around the interpretation of summarized results when the results of discordant studies are pooled in meta-analysis; this systematic review identified studies that are diverse particularly in regards to the type and dose of statin used and the duration of pre-operative therapy. There is currently no data available regarding type, dose, or duration of statin therapy in regards to modification of either post-operative clinical outcomes or the inflammatory response. The current body of evidence reviewed does not provide adequate data to examine directly treatment regimen to make any final conclusions on this topic. Lastly, small meta-analyses like those published here need to be regarded with caution even in the presence of statistically significant results.

Given that the existing RCTs relating to the current topic have important scientific and methodological limitations, including smaller sized samples, the importance of this systematic review lies in its identification of the gaps existing in the literature. In addition, the exploratory nature of the meta-analyses undertaken in this review provides a plausible estimate of effect that can be tested in subsequent studies.

## Conclusions

Although the RCT evidence may suggest a reduction in post-CPB inflammation by statin therapy, the evidence is not definitive due to significant limitations. Several of the trials were not methodologically rigorous and statin intervention was highly variable in this small number of studies. This systematic review demonstrates that there is a significant gap that exists in the current literature in regards to the potential anti-inflammatory effect of statin therapy prior to CPB. Further well-designed RCTs would help fill this gap and guide a rational development and use of interventions aimed at improving clinical outcomes post-CPB. There are two ongoing RCTs with large target sample sizes (over 600 patients in total), which will hopefully contribute some further evidence to this topic.

## Key messages

• Pre-operative statin may decrease inflammatory markers following CPB.

• Meta-analysis demonstrates benefit with the use of statin to reduce the post-operative level of IL-6, IL-8, CRP, and TNF-α.

• Given that the trial number is very small and some of the studies methodologically weak, the results need to be regarded with caution even in the presence of statistically significant results.

• Further well-designed and adequately powered trials are needed.

## Abbreviations

CABG: coronary artery bypass graft; CENTRAL: The Cochrane Central Register of Controlled Trials; CI: confidence interval; CPB: cardiopulmonary bypass; hsCRP: high sensitivity C-reactive protein; ICAM-1: intercellular adhesion molecule-1; IL: interleukin; NNT: number needed to treat; RCT: randomized controlled trials; RD: risk difference; RR: risk ratio; sP-selectin: soluble P-selectin; TNF-α: tumor necrosis factor-alpha; WMD: weighted mean difference.

## Competing interests

The authors declare that they have no competing interests.

## Authors' contributions

CM conceived of the study, designed the protocol, carried out the search, participated in trial selection, assessed methodological quality, extracted the data, carried out the data analysis and drafted the manuscript. MZ participated in trial selection, assessed methodological quality, extracted the data, and helped draft the manuscript. PG participated in trial selection and assessment of methodological quality. All authors read and approved the final manuscript.

## Supplementary Material

Additional file 1 A word file listing the details of the search strategy used. Click here for file

Additional file 2 A word file containing a series of tables providing the characteristics of excluded studies, studies awaiting assessment, and ongoing studies. Click here for file
