# Supplementary material for: Statin prophylaxis and inflammatory mediators following cardiopulmonary bypass: a systematic review
Source: Crit Care. 2009 Oct 20;13(5):R165. doi: 10.1186/cc8135 (PMC2784396; doi:10.1186/cc8135)
Supplement: Additional file 1 — A word file listing the details of the search strategy used. [file cc8135-S1.doc]

**Additional File 1: Details of search strategy**

**Search strategy: CENTRAL**

1. CARDIOPULMONARY BYPASS

2. cardiopulmonary next bypass

3. heart next bypass

4. OPEN HEART SURGERY

5. heart next surg

6. cardiac next surg

7. CORONARY ARTERY BYPASS

8. CABG

9. coronary near surg

10. coronary near bypass

11. CARDIAC SURGERY PROCEDURES

12. 1 or 2 or 3 or 4 or 5 or 6 or 7 or 8 or 9 or 10 or 11

13. HYDROXYMETHYLGLUTARYL-COA REDUCTASE INHIBITORS

14. statin*

15. atorvastatin

16. lovastatin

17. pitavastatin

18. pravastatin

19. rosuvastatin

20. simvastatin

21. cerivastatin

22. dalvastatin

23. fluindostatin

24. fluvastatin

25. mevinolin*

26. monacolin*

27. pravachol

28. lipex

29. lipitor

30. zocor

31. lescol

32. mevacor

33. baycol

34. 13 or 14 or 15 or 16 or 17 or 18 or 19 or 20 or 21 or 22 or 23 or 24 or 25 or

26 or 27 or 28 or 29 or 30 or 31 or 32 or 33

35. 12 and 34

**Search strategy: MEDLINE (OvidSP_UI01.00.02) 1966 to 2009**

1. exp extracorporeal circulation/

2. exp heart-lung machine/

3. "cardiopulmonary bypass".ti,ab.

4. "extracorporeal circulation".ti,ab.

5. "heart-lung machine".ti,ab.

6. "heart lung machine".ti,ab.

7. exp Cardiac Surgical Procedures/

8. "coronary artery bypass".ti,ab.

9. "thoracic surgery".ti,ab.

10. "cardiac surgery".ti,ab.

11. or/1-10

12. exp Hydroxymethylglutaryl-CoA Reductase Inhibitors/

13. Hydroxymethylglutaryl$.tw.

14. HMG CoA$.tw.

15. statin$.tw.

16. exp Anticholesteremic Agents/

17. 12 or 13 or 14 or 15 or 16

18. 11 and 17

19. randomized controlled trial.pt.

20. clinical trial.pt.

21. randomi?ed.ti,ab.

22. placebo.ti,ab.

23. dt.fs.

24. randomly.ti,ab.

25. trial.ti,ab.

26. groups.ti,ab.

27. or/19-26

28. animals/

29. humans/

30. 28 not (28 and 29)

31. 27 not 30

32. 31 and 18

**Search strategy: Embase (OvidSP_UI01.00.02) 1988 to 2009**

1. exp cardiopulmonary bypass/

2. "cardiopulmonary bypass".tw.

3. "heart surgery".tw.

4. "cardiac surgery".tw.

5. heart surgery/ or coronary artery surgery/ or coronary artery bypass graft/ or coronary artery bypass surgery/ or heart valve surgery/

6. "coronary artery bypass".tw.

7. or/1-6

8. exp Hydroxymethylglutaryl-CoA Reductase Inhibitors/

9. Hydroxymethylglutaryl$.tw.

10. HMG CoA$.tw.

11. statin$.tw.

12. exp Anticholesteremic Agents/

13. 8 or 9 or 10 or 11 or 12

14. exp clinical trial/

15. randomi?ed.ti,ab.

16. placebo.ti,ab.

17. dt.fs.

18. randomly.ti,ab.

19. trial.ti,ab.

20. groups.ti,ab.

21. or/14-20

22. animal/

23. human/

24. 22 not (22 and 23)

25. 21 not 24

26. 7 and 13 and 25
