# Supplementary material for: Statin prophylaxis and inflammatory mediators following cardiopulmonary bypass: a systematic review
Source: Crit Care. 2009 Oct 20;13(5):R165. doi: 10.1186/cc8135 (PMC2784396; doi:10.1186/cc8135)
Supplement: Additional file 2 — A word file containing a series of tables providing the characteristics of excluded studies, studies awaiting assessment, and ongoing studies. [file cc8135-S2.doc]

**Additional File 2: Characteristics of excluded studies, studies awaiting assessment, and ongoing studies**.

## Characteristics of excluded studies

| Study | Reason for exclusion |
| --- | --- |
| Brull et al. | Non-randomized trial; prospective cohort study |
| Chello et al. (2005) | Randomized Trial - primary outcome measurement of vascular function; Did not consider post-operative level of inflammatory markers or outcomes similar to primary and secondary outcomes of interest for the review. |
| Chello et al. (2003) | Non-randomized trial; prospective cohort study |
| Coccia et al. | Randomized Trial - primary outcome measurement erythrocyte membrane fluidity; Did not consider post-operative level of inflammatory markers or outcomes similar to primary and secondary outcomes of interest for the review. |
| Liakopoulos et al. | Non-randomized trial; prospective cohort study |
| Morawietz et al. | Randomized trial - primary outcome measurement anti-atherosclerotic endothelial expression quotient; did not consider post-operative level of inflammatory markers or outcomes similar to primary and secondary outcomes of interest for the review. |
| Pasceri et al. | Randomized Trial - primary outcome considered myocardial damage during intervention; did not consider post-operative level of inflammatory markers or outcomes similar to primary and secondary outcomes of interest for the review. |
| Wang et al. 2008 | Non-randomized trial; prospective cohort study |

##

## Characteristics of studies awaiting classification

| Nakamura 2006 | |
| --- | --- |
| **Methods** | Randomized trial |
| **Participants** | Thirty one adults scheduled for elective CABG (CPB or off-pump bypass)  Statin group n=15, age, mean 60, SD 13; control group n=16, age, mean 63, SD 8.  11/31 received CPB |
| **Interventions** | Atorvastatin 10 mg plus aspirin 100mg versus aspirin 100mg alone. Duration and timing of therapy not clear. |
| **Outcomes** | Inflammatory response, platelet activity, and coagulation activity. |
| **Notes** | No data or summary results published in article for group of people receiving CABG with CPB. Investigator was contacted regarding access to this data but no response. |

**CABG** – coronary artery bypass graft; **CPB** – cardiopulmonary bypass; **SD** – standard deviation;

## Characteristics of ongoing studies

| Bellomo 2008 | |
| --- | --- |
| **Study name** | Atorvastatin for renal protection after CPB |
| **Registration number** | ACTRN12606000405516 (Australian New Zealand Clinical Trials Registry) |
| **Methods** | Randomized controlled trial. Target sample size 102 |
| **Participants** | Patients having elective cardiac surgery requiring CPB; male and female; age minimum 18 years; more than 1 risk factor for renal dysfunction |
| **Interventions** | Atorvastatin 40 mg starting morning of surgery and the same dose continued for 3 days post-operatively |
| **Outcomes** | Acute kidney injury, systemic markers of oxidative stress and inflammation, adverse events. |
| **Starting date** | December 2006 |
| **Contact information** | Rinaldo.bellomo@austin.org.au |
| **Notes** | 82 randomized as of May 16, 2009, with expected recruitment completion end 2009 (personal communication) |

**CPB** – cardiopulmonary bypass

| Billings 2009 | |
| --- | --- |
| **Study name** | Short-term atorvastatin's effect on acute kidney injury following cardiac surgery |
| **Registration number** | NCT00791648 (ClinicalTrials.gov) |
| **Methods** | Randomized controlled trial. Target sample size 540 |
| **Participants** | Patients undergoing open heart surgery; male and female; age minimum 18 years; estimated glomerular filtration <90 mls/min/1.73m2 |
| **Interventions** | Atorvastatin 80 mg daily 3-7 days prior to open heart surgery until discharge or Atorvastatin 80 mg daily the day of open heart surgery and post-operative day 1 compared to placebo daily 3-7 days prior to open heart surgery until discharge or placebo daily day of open heart surgery and post-operative day 1 |
| **Outcomes** | Renal function, neurocognitive outcomes, plasma markers of inflammation |
| **Starting date** | April 2009 |
| **Contact information** | frederic.t.billings@vanderbilt.edu |
